# Mutations in the U4 snRNA gene *RNU4-2* cause one of the most prevalent monogenic neurodevelopmental disorders

**DOI:** 10.1038/s41591-024-03085-5

**Published:** 2024-05-31

**Authors:** Daniel Greene, Chantal Thys, Ian R. Berry, Joanna Jarvis, Els Ortibus, Andrew D. Mumford, Kathleen Freson, Ernest Turro

**Affiliations:** 1 Department of Medicine, University of Cambridge, Cambridge, UK; 2 Department of Genetics and Genomic Sciences, Icahn School of Medicine at Mount Sinai, New York, NY USA; 3 Department of Cardiovascular Sciences, Center for Molecular and Vascular Biology, KU Leuven, Leuven, Belgium; 4 NHS South West Genomic Laboratory Hub, Southmead Hospital, Bristol, UK; 5 NHS South West Genomic Medicine Service Alliance, Bristol, UK; 6 Clinical Genetics Unit, Birmingham Women’s Hospital, Birmingham, UK; 7 Department of Development and Regeneration, KU Leuven, Leuven, Belgium; 8 Paediatric Neurology Department, University Hospitals of KU Leuven, Leuven, Belgium; 9 Bristol Medical School, University of Bristol, Bristol, UK; 10 Mindich Child Health and Development Institute, Icahn School of Medicine at Mount Sinai, New York, NY USA; 11 Charles Bronfman Institute for Personalized Medicine, Icahn School of Medicine at Mount Sinai, New York, NY USA

**Keywords:** Neurodevelopmental disorders, Disease genetics

## Abstract

Most people with intellectual disability (ID) do not receive a molecular diagnosis following genetic testing. To identify new etiologies of ID, we performed a genetic association analysis comparing the burden of rare variants in 41,132 noncoding genes between 5,529 unrelated cases and 46,401 unrelated controls. *RNU4-2*, which encodes U4 small nuclear RNA, a critical component of the spliceosome, was the most strongly associated gene. We implicated de novo variants among 47 cases in two regions of *RNU4-2* in the etiology of a syndrome characterized by ID, microcephaly, short stature, hypotonia, seizures and motor delay. We replicated this finding in three collections, bringing the number of unrelated cases to 73. Analysis of national genomic diagnostic data showed *RNU4-2* to be a more common etiological gene for neurodevelopmental abnormality than any previously reported autosomal gene. Our findings add to the growing evidence of spliceosome dysfunction in the etiologies of neurological disorders.

## Abstract (web summary)

Mutations in a small noncoding gene called *RNU4-2* are responsible for one of the most common neurodevelopmental disorders that is mediated by a single gene.

## Main

Although 1,427 genes have been identified confidently as etiological for intellectual disability (ID)^1^, most ID cases remain unexplained following genetic testing^2^. All but nine of the 1,427 known genes are protein coding^3^, in part because the largest genetic studies of ID have used whole-exome sequencing (WES)^2^, which typically omits noncoding genes. To identify noncoding etiologies of ID, we conducted a genetic association analysis using whole-genome sequencing (WGS) data on 77,539 participants enrolled in the 100,000 Genomes Project (100KGP). This study included 29,741 probands and 4,782 affected relatives assigned by expert clinicians to one or more of 220 ‘Specific Disease’ classes encompassing a wide range of pathologies^4^. The remaining 43,016 participants were unaffected relatives. We built a Rareservoir database of genotypes and phenotypes^4^ for all the study participants and applied the BeviMed genetic association method^5^ to compare rare variant genotypes in 41,132 noncoding genes between 5,529 unrelated cases assigned to the Specific Disease class ID and 46,401 unrelated participants outside of that class.

**Fig. 1:**
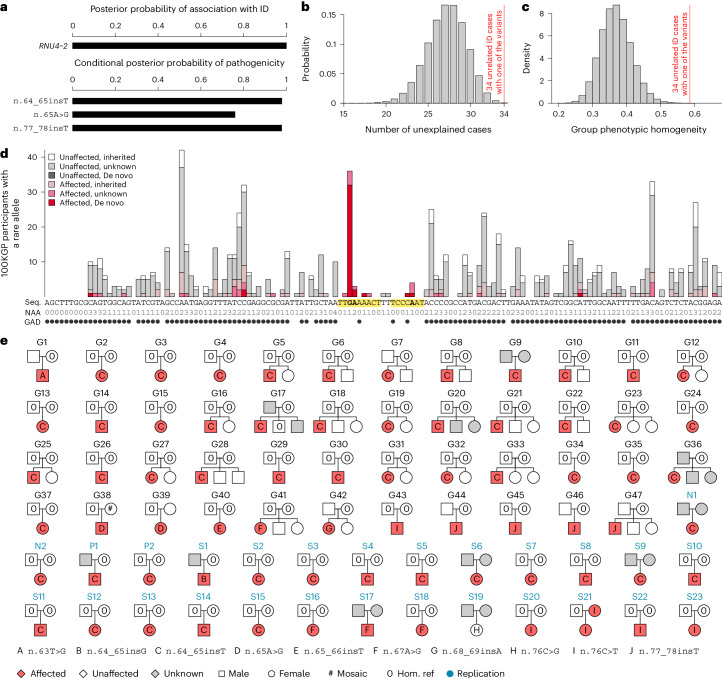
Discovery and replication of *RNU4-2* as an etiological gene for a novel neurodevelopmental disorder. **a**, BeviMed PPA between *RNU4-2* and the 100KGP Specific Disease ID. All other noncoding genes had a PPA < 0.5. Three *RNU4-2* variants had a conditional PPP > 0.5. **b**, Probability distribution for the number of unexplained cases among 34 randomly selected ID cases in the 100KGP. The actual number of unexplained ID cases (34) among the 34 ID cases with one of the three *RNU4-2* variants having a PPP > 0.5 is indicated with a red line. **c**. Distribution of phenotypic homogeneity scores (Methods) for randomly selected sets of 34 participants chosen from the 4,468 HPO-coded, unexplained, unrelated ID cases. The actual score for the 34 ID cases with one of the three *RNU4-2* variants having a PPP > 0.5 is indicated with a red line. **d**, For each of the 141 bases of the *RNU4-2* gene (Seq.), the number of participants with a rare allele at that position on the cDNA, stratified by affection status and inheritance information of the carried rare allele. The bases corresponding to the three variants with a PPP > 0.5 are in bold. These three bases and adjacent bases for which no unaffected participants carry a rare allele are highlighted. At each base, the number of distinct rare alternate alleles (NAA) observed in the 100KGP is shown. Underneath, the presence of an alternate allele in gnomAD is indicated by a filled circle (GAD). **e**, Pedigrees for participants with a rare alternate allele at one of the highlighted bases. Pedigrees used for discovery have a ‘G’ prefix and are labeled in black. NBR, 100KGP Pilot and GMS pedigrees used for replication have an ‘N’, ‘P’ or ‘S’ prefix, respectively, and are labeled in blue. The affection status for pedigree members who were not study participants was obtained from pedigree tables, except where the proband was assigned to a Specific Disease that was unrelated to neurodevelopmental disorders despite having an NDA (this applies to the nine grayed out members of G9, G17, G20 and G36). One variant was called homozygous for the reference allele in a parent but found to be mosaic by inspection of aligned WGS reads (#).

We identified an extremely strong dominant association between rare variants in *RNU4-2* and the risk of ID (posterior probability of association (PPA) ≈ 1, log Bayes factor = 55). *RNU4-2* is one of the genes encoding the U4 small nuclear RNA (snRNA) component of the small nuclear ribonuculeoprotein (snRNP) U4, which in turn is one of the five snRNPs of the major spliceosome. The association was much stronger than that for any other noncoding gene (all other PPAs < 0.5). Conditional on the association, three variants, observed among 34 ID cases, had a BeviMed posterior probability of pathogenicity (PPP) > 0.5 (Fig. 1a). None of the 34 cases were previously solved through an explanatory pathogenic (P) or likely pathogenic (LP) variant, representing a significant depletion compared to the 20.1% of ID cases overall explained through the 100KGP diagnostic pipeline (*P* = 4.86 × 10^−4^, one-sided binomial test; Fig. 1b). Based on Human Phenotype Ontology (HPO) terms assigned to 100KGP study participants, the 34 ID cases were phenotypically more homogeneous than expected by chance (*P* = 2.33 × 10^−5^, one-sided permutation test; Fig. 1c), further suggesting that the association was causal. In addition to the 34 ID cases, a further eight participants outside the Specific Disease class ID also carried one of the three variants. Seven of these had the HPO term ‘Neurodevelopmental abnormality’ (NDA) and one had related ICD10 (International Classification of Diseases, tenth revision) codes, such as ‘Unspecified intellectual disabilities’, providing internal replication among participants treated as controls in the association analysis. To account for variations in Specific Disease assignment and phenotype coding, we hereafter consider 100KGP participants with the NDA HPO term or an ‘Intellectual disabilities’ ICD10 code to be affected with a neurodevelopmental disorder.

**Extended Data Fig. 1:**
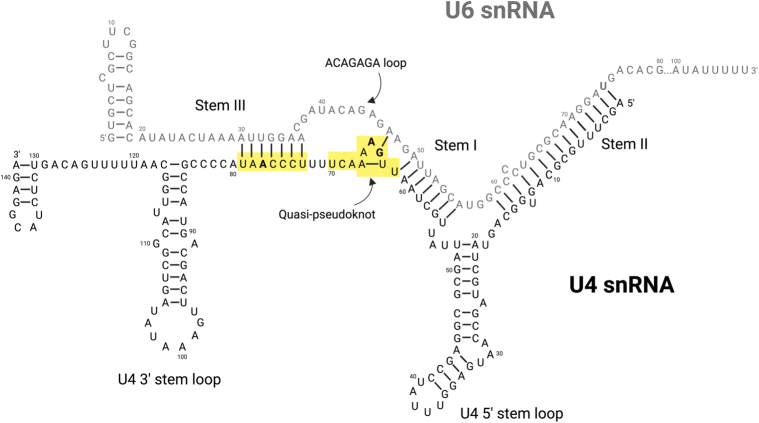
Organization of the U4/U6 snRNA duplex within the tri-snRNP U4/U6.U5. Human U4 snRNA is stabilized by three intramolecular stem loop motifs (ribonucleotides 20–52, 85–117 and 127–144) and maintains an inhibitory interaction with U6 snRNA through extensive intermolecular pairings involving U4 ribonucleotides within stem I (56–61), stem II (1–16), stem III (73–79) and the U4 quasi-pseudoknot (62 and 64). This region is further stabilized by interactions between U4 ribonucleotides 68–70 and the RNA-binding protein 42 (RBM42)^8,25,26^ and with more extensive contacts between this region and U4/U6.U5 tri-snRNP-associated protein 1 (SNUT1)^27^ and U4/U6.U5 snRNP 27 kDa protein (SNRNP-27K)^28^. The U4 stem III, quasi-pseudoknot and associated RNA binding proteins determine the orientation of the U6 snRNA ACAGAGA loop, ensuring its accessibility for interaction with the 5′ splice site of target introns that is necessary for the catalytic activation of the spliceosome complex^8^. The variants associated with neurodevelopmental disorder lie in the central region of the U4/U6 snRNA duplex (yellow boxes; bolded ribonucleotides represent those harboring variants with a PPP > 0.5), are predicted to disrupt Watson-Crick interactions within U4 stem III (n.76 C > T and n.77_78insT) or to disrupt configuration of the U4 quasi-pseudoknot (n.63 T > G; n.64_65insG; n.64_65insT; n65A>G; n.65_66insT; n.67 A > G and n.68_69insA) thereby de-stabilizing U4 snRNA interactions with U6 snRNA and with RNA binding proteins that are necessary for correct spliceosome function. The image represents the predicted U4/U6 duplex secondary structure with ribonucleotide numbering according to the canonical *RNU4-2* and *RNU6-1* reference sequences ENST00000365668.1 and ENST00000383898.1 respectively.

**Extended Data Fig. 2:**
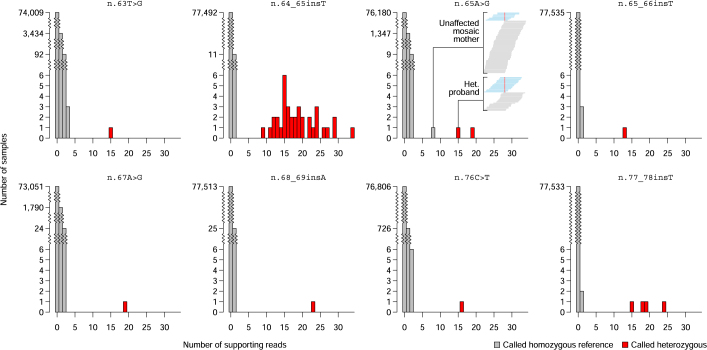
Mosaicism analysis. For each of the eight rare variants carried by 100KGP participants in the highlighted region of Fig. 1c, truncated bar charts showing the distribution of the number of reads supporting the alternate allele. The participants called heterozygous for a given *RNU4-2* rare variant are represented in red, while other participants are represented in gray. The embedded window in the panel for variant n.65 A > G shows the read pileups at this position in the heterozygous proband of pedigree G38 and his mother, who had a homozygous reference genotype call but is likely mosaic. The reads supporting the reference allele are in blue and those supporting the variant allele are in gray.

Five additional variants within a few base pairs of the three variants with a PPP > 0.5 were also present exclusively in affected participants, forming two contiguous regions (n.62–70 and n.73–79) within which no unaffected participants had a rare variant. These regions were embedded within a genomic locus markedly depleted of variation in gnomAD^6^ (Fig. 1d), consistent with the effect of purifying selection. Analysis of published secondary structure data of the U4 snRNA revealed that one region maps to a quasi-pseudoknot interaction between U4 and U6 while the other maps to an interaction between U4 and U6 called stem III^7^ (Extended Data Fig. 1). Of the 47 rare allele instances in the regions, which we observed in 47 cases, 37 were determined to be de novo on the basis of genotype calls in the respective parents, while 10 had unknown inheritance because of missing genotypes from one or both of the corresponding parents (Fig. 1d,e). The most common variant, n.64_65insT, was observed as a de novo mutation in 33 different families. Intriguingly, the G at position 64 of U4 snRNA is thought to contribute to the stability of the ACAGAGA loop of U6 snRNA, which binds 5′ splice sites and induces splicing after U4–U6 unwinding^8^. Inspection of read alignments across the 77,539 100KGP participants to assess the quality of genotyping revealed that one of the parents was mosaic for n.65A>G, while the remainder had confident heterozygous or homozygous reference genotype calls (Extended Data Fig. 2).

We sought to replicate our findings in three additional collections: the National Institute for Health and Care Research (NIHR) BioResource for Rare Diseases (NBR)^9^ (which includes 731 NDA-coded cases out of 7,388 participants enrolled for research), the 100KGP Pilot Project (which includes 291 NDA-coded cases out of 4,054 100KGP participants enrolled in a pilot phase before the 100KGP’s main program was established) and the UK’s Genomic Medicine Service (GMS) (which includes 5,527 NDA-coded cases out of 25,288 participants sequenced prospectively through the UK’s National Health Service (NHS)). Across these three collections, we identified a further 27 probands with a rare variant within the two regions of interest, of whom 25 were NDA coded, one was coded with ‘Abnormal brain morphology’ terms such that NDA could be inferred, and one was unaffected (Fig. 1e). In total, 19 of the 26 affected probands acquired the variant de novo, six acquired the variant with inheritance that was unknown because of a lack of parental genotype data and one case inherited variant n.76C>T from an affected mother. The n.76C>T variant is the only one among the 74 pedigrees that is present in gnomAD, where it has an allele count of one in 152,108. While we observed this variant as a de novo mutation in four families, the inheritance in one family and the observation in gnomAD suggest that it may cause a less severe phenotype in some cases than the other variants we identified, particularly the predominant variant n.64_65insT. Moreover, variant n.76C>G was observed in one case without NDA, further suggesting that certain mutations at nucleotide position 76 may have a limited or benign effect.

**Fig. 2:**
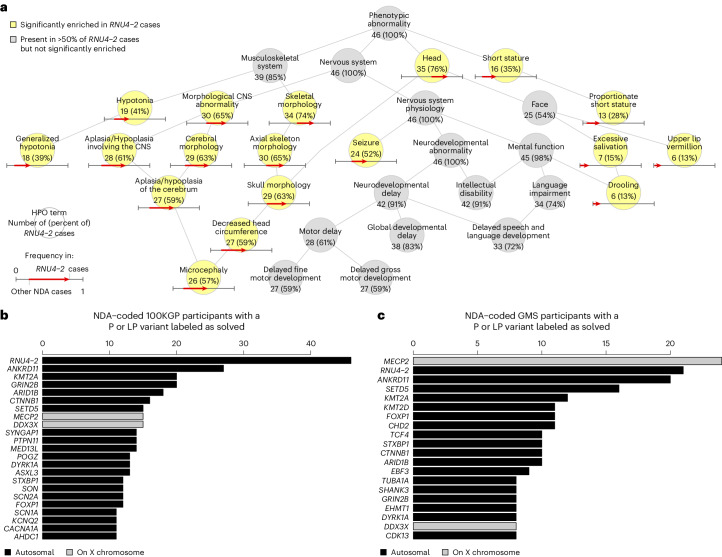
Phenotypic characterization and prevalence of a novel neurological disorder. **a**, Graph showing the is-a relationships among HPO terms present in at least half of the 46 NDA-coded *RNU4-2* cases identified or significantly enriched among these 46 cases relative to 9,112 other unrelated NDA-coded participants of the 100KGP. Terms are shortened to remove ‘Abnormality of (the)’ or ‘Abnormal’ for conciseness. The significantly overrepresented terms are highlighted. For each term, the number of cases with the term and the percentage that number represents out of 46 is shown. For each overrepresented term, the proportion of NDA-coded participants that are not *RNU4-2* cases with the term and the proportion of NDA-coded *RNU4-2* cases with the term are represented as the base and the head of an arrow, respectively. **b**, Of the 9,112 NDA-coded cases in the 100KGP, the number solved through P or LP variants in a gene, provided at least 11 cases were diagnosed. In the case of *RNU4-2*, the number of NDA-coded cases with a rare variant in the highlighted region of Fig. 1d is shown instead of the number solved with P or LP variants. **c**, Of the 5,527 NDA-coded cases in the GMS, the number solved through P or LP variants in a gene, provided at least eight cases were diagnosed. In the case of *RNU4-2*, the number of NDA-coded cases with a rare variant in the highlighted region of Fig. 1d is shown instead of the number solved with P or LP variants.

To further characterize this new syndrome, we analyzed the HPO terms of the 46 100KGP cases annotated with the NDA term (one of the 47 cases had consistent ICD10 codes but was erroneously not annotated with the NDA HPO term). Of these 46 cases, 91% had the ID term, 91% had ‘Neurodevelopmental delay’ and 61% had ‘Motor delay’, broadly in line with general term frequencies across ID cases (Fig. 2a). However, several terms were significantly overrepresented among the 46 *RNU4-2* cases, including ‘Microcephaly’ in 57% (versus 18% in other NDA-coded cases, Bonferroni-adjusted *P* = 3.23 × 10^−7^), ‘Drooling’ in 13% (versus 1%, *P* = 6.93 × 10^−4^), ‘Proportionate short stature’ in 28% (versus 7%, *P* = 7.60 × 10^−4^), ‘Generalized hypotonia’ in 39% (versus 13%, *P* = 8.08 × 10^−4^), ‘Seizure’ in 52% (versus 27%, *P* = 3.13 × 10^−2^) and ‘Abnormality of upper lip vermillion’ in 13% (versus 2%, *P* = 4.00 × 10^−2^) (Fig. 2a). No terms were significantly underrepresented in the *RNU4-2* cases.

To assess the prevalence of this new disorder, we compared the number of NDA-coded *RNU4-2* cases in the 100KGP (46 of 9,112 NDA-coded cases, 0.50%) with the number of solved cases with P or LP variants in other etiological genes for NDA. *RNU4-2* was the most prevalent etiology for NDA in the 100KGP (Fig. 2b). As the 100KGP was subject to genetic prescreening, we also assessed the prevalence among persons undergoing diagnostic WGS through the UK GMS, which prescreens only for aneuploidy, copy-number variants, tandem repeats and abnormal methylation. In the GMS, *RNU4-2* was the second most prevalent etiology for NDA (21 of 5,527 NDA-coded cases, 0.38%) after *MECP2* (24 cases), a long-established etiological X-linked gene for Rett syndrome^10^ (Fig. 2c).

**Extended Data Fig. 3:**
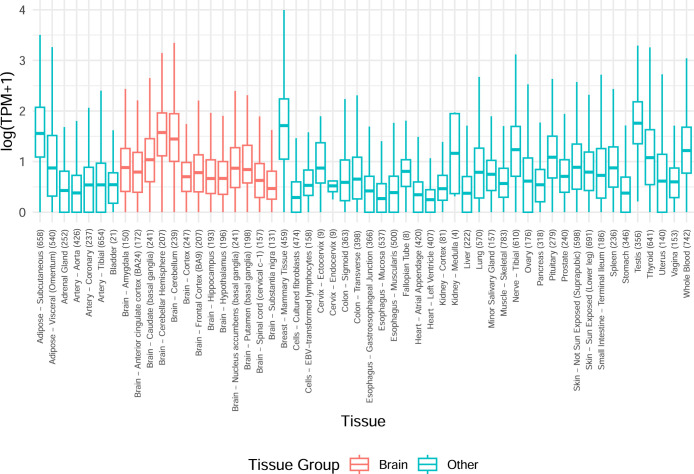
Expression of the gene encoding the U4 snRNA, *RNU4-2*, across tissues. Boxplot of *RNU4-2* log expression across adult tissues subjected to RNA-seq by the GTEx Consortium^29^. The number of samples corresponding to each tissue type is shown in brackets. Brain tissue types are highlighted. TPM: transcripts per million. The lower, center and upper edges of the boxes respectively indicate the lower quartile, median and upper quartiles. Whiskers are drawn up to the most extreme points that are less than 1.5 times the interquartile range away from the nearest quartile.

Splicing of eukaryotic pre-mRNA is catalyzed by large macromolecular complexes termed spliceosomes that comprise snRNPs and additional proteins^11^. The major spliceosome assembles on pre-mRNA through the sequential association of five snRNPs (U1, U2, U4, U5 and U6), each containing a unique snRNA, and a total of approximately 100 additional proteins^12^. Assembly of the major spliceosome is initiated by binding of U1 and U2 snRNPs to the pre-mRNA strand. Thereafter, the preassembled U4–U6·U5 tri-snRNP is recruited to form the inactive pre-B complex. The interaction between U4 and U6 snRNAs within the tri-snRNP (Extended Data Fig. 1) contributes to the structural stability of the pre-B complex, particularly of the critical ACAGAGA loop of U6 snRNA^13^ that helps ensure the fidelity of the interaction with the intron. Activation of the major spliceosome is initiated by PRP28, which transfers the 5′ splice site to U5 snRNP, thereby forming the major spliceosome B complex. This is followed by disassembly of the U4–U6 duplex by the helicase SNRNP200 (also known as BRR2), which is followed by pairing with U2 snRNP at the 3′ splice site and complete removal of the intron^8^. Many components of the major spliceosome are ubiquitously expressed, including *RNU4-2* (Extended Data Fig. 3). However, some interacting protein components show tissue-specific expression, which might explain why pathogenic variants in genes encoding some of these components cause defects (spliceosomeopathies) that are restricted to subsets of cell lineages^14^. The same applies to genes encoding snRNAs of the minor spliceosome. Compound heterozygous and homozygous variants in *RNU4ATAC* and *RNU12* can cause recessive conditions restricted to certain organ systems in certain persons, albeit with apparently fully penetrant ID^15–18^. Before the present work, none of the five snRNAs of the major spliceosome were implicated in a human disorder. Very recently, biallelic loss-of-function variants in *WBP4*, which encodes a protein component of the major spliceosome, were found to cause a neurodevelopmental syndrome with remarkably similar phenotypes to those we describe here, including hypotonia, global developmental delay, severe ID, brain abnormalities and musculoskeletal defects^19^. WW domain-binding protein 4 (WBP4) delays the U4–U6 unwinding activity of SNRNP200 and thereby spliceosome activation^20^. Cryo-EM structures of the pre-B complex suggest that the quasi-pseudoknot promotes interaction between 5′ splice sites and U6 snRNA’s ACAGAGA loop to bring about SNRNP200 relocation in U4 snRNA, enabling SNRNP200 to unwind U4–U6 and trigger splicing activation^8^. Given that the predominant variant, n.64_65insT, could disrupt the G64(U4)–A47(U6) interaction supporting the quasi-pseudoknot structure, a delay in U4–U6 unwinding activity similar to that observed in WBP4 deficiency might be the underlying etiological mechanism of this newly described syndrome.

## Methods

### Enrollment criteria for the ID class in the 100KGP

The enrollment criteria for all specific diseases in the 100KGP are available from the 100KGP website^21^. The provided criteria for ID are reproduced here verbatim. “ID inclusion criteria. Moderate to severe/profound ID disproportionate to parental IQ unless the family history is consistent with an X-linked disorder; congenital onset; developmental delay; ± clinical features suggestive of a specific syndrome; metabolic causes have been excluded. ID exclusion criteria. Antenatal history suggestive of non-genetic cause, proven congenital or neonatal infections; known genetic cause already identified; microarray analysis abnormal and clearly pathogenic*.* Prior genetic testing guidance. Results should have been reviewed for all genetic tests undertaken, including disease-relevant genes in WES data; the patient is not eligible if they have a molecular diagnosis for their condition; genetic testing should continue according to routine local practice for this phenotype regardless of recruitment to the project; results of these tests must be submitted through the ‘Genetic investigations’ section of the data capture tool to allow comparison of WGS with current standard testing. ID prior genetic testing genes. Testing of the following genes should be carried out prior to recruitment where this is in line with current local practice: for syndromes where the cause of disease is 1–2 genes these need to be excluded before recruitment, for example, for Kabuki syndrome, *KMT2D* and *KDM6A* should have been tested.”

### Genetic association analysis

We constructed a Rareservoir database^4^ in the Genomics England Research Environment containing rare variants extracted from 77,539 consented participants in each of the 41,132 noncoding canonical transcripts listed in Ensembl version 104. We annotated the participants with Specific Disease classes and HPO terms. We imputed the ID HPO term in cases in the ID class wherever the term had been omitted. We annotated and filtered the variants as previously described^4^. We applied the BeviMed^5^ association test to each noncoding gene comparing unrelated participants in the ID class with unrelated participants outside of that class. The set of cases was constructed by selecting one case from each pedigree containing at least one person assigned to the ID class and the set of controls was formed by taking the intersection of the maximal set of unrelated participants provided by the 100KGP with participants not related to any of the cases.

Using BeviMed, we performed a Bayesian comparison of three models:

1. No association (prior probability 0.99)
2. Dominant association, taking variants with a probabilistic minor allele frequency (pMAF)^9^ < 0.01% (prior probability 0.005)
3. Recessive association, taking variants with a pMAF < 0.1%, (prior probability 0.005).

Thus, the overall prior probability of association was 0.01. The hyperparameters were set to the default values given in the literature^5^. The PPA was the sum of the posterior probabilities of models 2 and 3. The analysis was carried out using R 3.6.2, making use of packages Matrix 1.2–18, dplyr 0.8.5, bit64 0.9–7, bit 1.1–14, DBI 1.1.0, RSQLite 2.1.4 and BeviMed 5.7.

### Phenotypic similarity analysis

To determine whether a given subset of HPO-annotated, unrelated participants was more phenotypically homogeneous than expected by chance, we applied the following approach: We computed the information content (IC) of each HPO term as −log of its frequency. We chose Resnik’s^22^ similarity function *s* to compute the similarity between two terms *t*_1_ and *t*_2_: $s({t}_{1},{t}_{2})=\mathop{\rm{max}}\limits_{t\in {\rm{anc}}({t}_{1})\cap {\rm{anc}}({t}_{2})}{\rm{IC}}(t)$, where anc(*t*) denotes the union of term *t* and its ancestral nodes in the HPO graph. We defined the similarity *S* between two sets of terms *ϕ*_1_ and *ϕ*_2_ (for example, those attached to two study participants) using the best match average function^23^: $S({\phi }_{1},{\phi }_{2})=\frac{1}{2|{\phi }_{1}|}\sum _{{t}_{1}\in {\phi }_{1}}\mathop{\rm{max}}\limits_{{t}_{2}\in {\phi }_{2}}s({t}_{1},{t}_{2})+\frac{1}{2|{\phi }_{2}|}\sum _{{t}_{2}\in {\phi }_{2}}\mathop{\rm{max}}\limits_{{t}_{1}\in {\phi }_{1}}s({t}_{2},{t}_{1})$. We defined the phenotypic homogeneity of a group of size *k* as the mean pairwise similarity: $$h({\phi }_{1},{\phi }_{2},\ldots ,{\phi }_{k})={\left(\begin{array}{c}k\\ 2\end{array}\right)}^{-1}\mathop{\sum }\nolimits_{i=1}^{k-1}\mathop{\sum }\nolimits_{j=i+1}^{k}S({\phi }_{i},{\phi }_{j})$$. To determine whether the homogeneity of a group of size *k* was significantly greater than expected by chance, we selected sets of *k* participants at random and obtained a Monte Carlo *P* value as the proportion of random sets that had a homogeneity greater than or equal to the homogeneity of the group.

### Phenotypic characterization

To identify enriched or depleted HPO terms among the 46 NDA-annotated cases with *RNU4-2* variants in the regions of interest, compared to unrelated NDA-coded participants without *RNU4-2* variants, we computed *P* values of association using Fisher’s two-sided exact test. We only tested enrichment for terms attached to at least five of the 46 cases and that belonged to the set of nonredundant terms at each level of frequency among the cases. To account for multiple comparisons, we adjusted the *P* values by multiplying them by the number of tests. An adjusted *P* value < 0.05 was deemed statistically significant. To visualize both common and distinctive HPO terms for *RNU4-2* cases, we selected terms that were either statistically significant or present in at least 50% of the cases, removed redundant terms at each level of frequency among the 46 cases and arranged the terms along with a nonredundant set of ancestral terms as a directed acyclic graph of is-a relations. These analyses were conducted using the ontologyX R package^24^.

### Ethics

Participants of the 100KGP, the 100KGP Pilot Project and the GMS were enrolled to the National Genomic Research Library under a protocol approved by the East of England–Cambridge Central Research Ethics Committee (20/EE/0035). NBR participants were enrolled under a protocol approved by the East of England–Cambridge South Research Ethics Committee (13/EE/0325). The Ethics Committee of University Hospitals Leuven approved genetic and experimental studies of a pedigree enrolled to the NBR in Belgium (ML3580/S50025 and S63666). Only participants who provided written informed consent for their data to be used for research were included in the analyses.

### Reporting summary

Further information on research design is available in the Nature Portfolio Reporting Summary linked to this article.

## Online content

Any methods, additional references, Nature Portfolio reporting summaries, source data, extended data, supplementary information, acknowledgements, peer review information; details of author contributions and competing interests; and statements of data and code availability are available at 10.1038/s41591-024-03085-5.

### Supplementary information

Reporting Summary

## Data Availability

Genetic and phenotypic data for the 100KGP study participants, the 100KGP Pilot study participants and the GMS participants are available through the Genomics England Research Environment through application at https://www.genomicsengland.co.uk/join-a-gecip-domain. Data pertaining to WGS data were obtained from a merged variant call format file (VCF) for 77,539 100KGP participants, a merged VCF for 4,054 100KGP Pilot participants, single-genome VCFs for 25,289 GMS participants (v3) and single sample gVCFs for 13,037 NBR participants. HPO phenotype data were obtained from the ‘rare_diseases_participant_phenotype’ table (Main Program v13), ‘observation’ table (GMS v3) and ‘hpo’ table (Rare Diseases Pilot v3). Specific Disease class data were obtained from the ‘rare_diseases_participant_disease’ table (Main Program v13). ICD10 codes were obtained from the ‘hes_apc’ table (Main Program v13). Pedigree information was obtained from the ‘rare_diseases_pedigree_member’ table (Main Program v13), ‘referral_participant’ table (GMS v3) and ‘pedigree’ table (Rare Diseases Pilot v3). The explained or unexplained status of cases was obtained from the ‘gmc_exit_questionnaire’ tables (Main Program v18, GMS v3). Accession codes for NBR data are given in the literature^9^. CADD version 1.5 (https://cadd.gs.washington.edu/), gnomAD version 3.0 (https://gnomad.broadinstitute.org/) and Ensembl version 104 (http://may2021.archive.ensembl.org/index.html) were used for variant annotation. Data presented in this paper were requested from the Genomics England Airlock on April 2, 2024 at 11:08 p.m. British Summer Time (BST). The manuscript was submitted to the Genomics England Publication Committee on April 8, 2024 at 5:45 a.m. BST and approved for submission on April 11, 2024 at 12:24 p.m. BST.

## Code availability

Software packages rsvr 1.0, bcftools 1.16, samtools 1.9 and perl 5 were used to build the noncoding 100KGP Rareservoir. The Rareservoir software is available from https://github.com/turrogroup/rsvr. All R packages listed in the manuscript are available from the Comprehensive R Archive Network site (https://cran.r-project.org).

Expression data for *RNU4-2* were extracted from the file ‘GTEx_Analysis_2017-06-05_v8_RNASeQCv1.1.9_gene_tpm.gct.gz’ available from the GTEx Portal.

## References

[1] Martin, A. R. et al. PanelApp crowdsources expert knowledge to establish consensus diagnostic gene panels. *Nat. Genet.* **51**, 1560–1565 (2019). 31676867 10.1038/s41588-019-0528-2

[2] Wright, C. F. et al. Genomic diagnosis of rare pediatric disease in the United Kingdom and Ireland. *N. Engl. J. Med.* **388**, 1559–1571 (2023). 37043637 10.1056/NEJMoa2209046 PMC7614484

[3] Genomics England PanelApp. Available from https://panelapp.genomicsengland.co.uk (accessed on April 4, 2024), intellectual disability—microarray and sequencing (version 5.515).

[4] Greene, D. et al. Genetic association analysis of 77,539 genomes reveals rare disease etiologies. *Nat. Med.* **29**, 679–688 (2023). 36928819 10.1038/s41591-023-02211-z PMC10033407

[5] Greene, D., Richardson, S. & Turro, E. A fast association test for identifying pathogenic variants involved in rare diseases. *Am. J. Hum. Genet.* **101**, 104–114 (2017). 28669401 10.1016/j.ajhg.2017.05.015 PMC5501869

[6] Chen, S. et al. A genomic mutational constraint map using variation in 76,156 human genomes. *Nature* **625**, 92–100 (2024). 38057664 10.1038/s41586-023-06045-0 PMC11629659

[7] Jakab, G. et al. *Chlamydomonas* U2, U4 and U6 snRNAs. An evolutionary conserved putative third interaction between U4 and U6 snRNAs which has a counterpart in the U4_atac_–U6_atac_ snRNA duplex. *Biochimie* **79**, 387–395 (1997). 9352088 10.1016/S0300-9084(97)86148-2

[8] Charenton, C., Wilkinson, M. E. & Nagai, K. Mechanism of 5′ splice site transfer for human spliceosome activation. *Science* **364**, 362–367 (2019). 30975767 10.1126/science.aax3289 PMC6525098

[9] Turro, E. et al. Whole-genome sequencing of patients with rare diseases in a national health system. *Nature* **583**, 96–102 (2020). 32581362 10.1038/s41586-020-2434-2 PMC7610553

[10] Amir, R. E. et al. Rett syndrome is caused by mutations in X-linked *MECP2*, encoding methyl-CpG-binding protein 2. *Nat. Genet.* **23**, 185–188 (1999). 10508514 10.1038/13810

[11] Pan, Q. et al. Deep surveying of alternative splicing complexity in the human transcriptome by high-throughput sequencing. *Nat. Genet.* **40**, 1413–1415 (2008). 18978789 10.1038/ng.259

[12] Wilkinson, M. E., Charenton, C. & Nagai, K. RNA splicing by the spliceosome. *Annu. Rev. Biochem.* **89**, 359–388 (2020). 31794245 10.1146/annurev-biochem-091719-064225

[13] Nguyen, T. H. et al. The architecture of the spliceosomal U4/U6·U5 tri-snRNP. *Nature* **523**, 47–52 (2015). 26106855 10.1038/nature14548 PMC4536768

[14] Griffin, C. & Saint-Jeannet, J. P. Spliceosomopathies: diseases and mechanisms. *Dev. Dyn.* **249**, 1038–1046 (2020). 32506634 10.1002/dvdy.214 PMC8603363

[15] He, H. et al. Mutations in U4_atac_ snRNA, a component of the minor spliceosome, in the developmental disorder MOPD I. *Science* **332**, 238–240 (2011). 21474760 10.1126/science.1200587 PMC3380448

[16] Merico, D. et al. Compound heterozygous mutations in the noncoding RNU4ATAC cause Roifman Syndrome by disrupting minor intron splicing. *Nat. Commun.* **6**, 8718 (2015). 26522830 10.1038/ncomms9718 PMC4667643

[17] Farach, L. S. et al. The expanding phenotype of RNU4ATAC pathogenic variants to Lowry Wood syndrome. *Am. J. Med. Genet. A* **176**, 465–469 (2018). 29265708 10.1002/ajmg.a.38581 PMC6774248

[18] Elsaid, M. F. et al. Mutation in noncoding RNA RNU12 causes early onset cerebellar ataxia. *Ann. Neurol.* **81**, 68–78 (2017). 27863452 10.1002/ana.24826

[19] Engal, E. et al. Bi-allelic loss-of-function variants in *WBP4*, encoding a spliceosome protein, result in a variable neurodevelopmental syndrome. *Am. J. Hum. Genet.* **110**, 2112–2119 (2023). 37963460 10.1016/j.ajhg.2023.10.013 PMC10716347

[20] Henning, L. M. et al. A new role for FBP21 as regulator of Brr2 helicase activity. *Nucleic Acids Res.* **45**, 7922–7937 (2017). 28838205 10.1093/nar/gkx535 PMC5570060

[21] Devereau, A. 100,000 Genomes Project Rare Disease Eligibility Criteria (Genomics England, 2018); https://files.genomicsengland.co.uk/forms/Rare-Disease-Eligibility-Criteria.pdf

[22] Resnik, P. et al. Semantic similarity in a taxonomy: an information-based measure and its application to problems of ambiguity in natural language. *J. Artif. Intell. Res.* **11**, 95–130 (1999). 10.1613/jair.514

[23] Köhler, S. et al. Clinical diagnostics in human genetics with semantic similarity searches in ontologies. *Am. J. Hum. Genet.* **85**, 457–464 (2009). 19800049 10.1016/j.ajhg.2009.09.003 PMC2756558

[24] Greene, D., Richardson, S. & Turro, E. ontologyX: a suite of R packages for working with ontological data. *Bioinformatics* **33**, 1104–1106 (2017). 28062448 10.1093/bioinformatics/btw763 PMC5386138

[25] Agafonov, D. E. et al. Molecular architecture of the human U4/U6·U5 tri-snRNP. *Science* **351**, 1416–1420 (2016). 26912367 10.1126/science.aad2085

[26] Boesler, C. et al. A spliceosome intermediate with loosely associated tri-snRNP accumulates in the absence of Prp28 ATPase activity. *Nat. Commun.* **7**, 11997 (2016). 27377154 10.1038/ncomms11997 PMC4935976

[27] Sarka, K., Katzman, S. & Zahler, A. M.A role for SNU66 in maintaining 5′ splice site identity during spliceosome assembly. *RNA* **30**, 695–709 (2024). 38443114 10.1261/rna.079971.124 PMC11098459

[28] Leung, A. K., Nagai, K. & Li, J. Structure of the spliceosomal U4 snRNP core domain and its implication for snRNP biogenesis. *Nature* **473**, 536–539 (2011). 21516107 10.1038/nature09956 PMC3103711

[29] Aguet, F. et al. The GTEx Consortium atlas of genetic regulatory effects across human tissues. *Science* **369**, 1318–1330 (2020). 32913098 10.1126/science.aaz1776 PMC7737656

