# Supplementary material for: Mutations in the U4 snRNA gene RNU4-2 cause one of the most prevalent monogenic neurodevelopmental disorders
Source: Nat Med. 2024 May 31;30(8):2165–9. doi: 10.1038/s41591-024-03085-5 (PMC11333284; doi:10.1038/s41591-024-03085-5)
Supplement: Supplementary file 1 — Reporting Summary [file 41591_2024_3085_MOESM1_ESM.pdf]

nature portfolio

Corresponding author(s): Ernest Turro

Last updated by author(s): May 17, 2024



# Reporting Summary

Nature Portfolio wishes to improve the reproducibility of the work that we publish. This form provides structure for consistency and transparency in reporting. For further information on Nature Portfolio policies, see our Editorial Policies and the Editorial Policy Checklist.

## Statistics

For all statistical analyses, confirm that the following items are present in the figure legend, table legend, main text, or Methods section.

| n/a | Confirmed | |
|---|---|---|
| ☐ | ☒ | The exact sample size (*n*) for each experimental group/condition, given as a discrete number and unit of measurement |
| ☒ | ☐ | A statement on whether measurements were taken from distinct samples or whether the same sample was measured repeatedly |
| ☐ | ☒ | The statistical test(s) used AND whether they are one- or two-sided<br>*Only common tests should be described solely by name; describe more complex techniques in the Methods section.* |
| ☐ | ☒ | A description of all covariates tested |
| ☐ | ☒ | A description of any assumptions or corrections, such as tests of normality and adjustment for multiple comparisons |
| ☐ | ☒ | A full description of the statistical parameters including central tendency (e.g. means) or other basic estimates (e.g. regression coefficient) AND variation (e.g. standard deviation) or associated estimates of uncertainty (e.g. confidence intervals) |
| ☒ | ☐ | For null hypothesis testing, the test statistic (e.g. *F*, *t*, *r*) with confidence intervals, effect sizes, degrees of freedom and *P* value noted<br>*Give P values as exact values whenever suitable.* |
| ☐ | ☒ | For Bayesian analysis, information on the choice of priors and Markov chain Monte Carlo settings |
| ☒ | ☐ | For hierarchical and complex designs, identification of the appropriate level for tests and full reporting of outcomes |
| ☒ | ☐ | Estimates of effect sizes (e.g. Cohen's *d*, Pearson's *r*), indicating how they were calculated |

*Our web collection on statistics for biologists contains articles on many of the points above.*

## Software and code

Policy information about availability of computer code

| | |
|---|---|
| Data collection | samtools 1.9; bcftools 1.16; perl 5. |
| Data analysis | rsvr 1.0 (https://github.com/turrogroup/rsvr); bcftools 1.16; samtools 1.9; perl 5; R 3.6.2; R packages Matrix 1.2-18, dplyr 0.8.5, bit64 0.9-7, bit 1.1-14, DBI 1.1.0, RSQLite 2.1.4, BeviMed 5.7, ontologyIndex 2.12, ontologySimilarity 2.7, ggplot2 3.5.0. |

For manuscripts utilizing custom algorithms or software that are central to the research but not yet described in published literature, software must be made available to editors and reviewers. We strongly encourage code deposition in a community repository (e.g. GitHub). See the Nature Portfolio guidelines for submitting code & software for further information.

## Data

Policy information about availability of data

All manuscripts must include a data availability statement. This statement should provide the following information, where applicable:

- Accession codes, unique identifiers, or web links for publicly available datasets
- A description of any restrictions on data availability
- For clinical datasets or third party data, please ensure that the statement adheres to our policy

Genetic and phenotypic data for the 100KGP study participants, the 100KGP Pilot study participants and the GMS participants are available through the Genomics England Research Environment via the application at https://www.genomicsengland.co.uk/join-a-gecip-domain. Data pertaining to: WGS data were obtained from a merged variant call format file (VCF) for 77,539 100KGP participants, a merged VCF for 4,054 100KGP Pilot participants, single genome VCFs for 25,289 GMS

participants (v3) and single sample gVCFs for 13,037 NBR participants; HPO phenotype data from the 'rare_diseases_participant_phenotype' table (Main Programme v13), 'observation' table (GMS v3) and 'hpo' table (Rare Diseases Pilot v3); Specific Disease class data from the 'rare_diseases_participant_disease' table (Main Programme v13); ICD10 codes from the 'hes_apc' table (Main Programme v13); pedigree information from the 'rare_diseases_pedigree_member' table (Main Programme v13), 'referral_participant' table (GMS v3), and 'pedigree' table (Rare Diseases Pilot v3); explained/unexplained status of cases from the 'gmc_exit_questionnaire' tables (Main Programme v18, GMS v3). Accession codes for NBR data are given in ref.[PMID:32581362]. CADD v.1.5 (https://cadd.gs.washington.edu/), gnomAD v.3.0 (https://gnomad.broadinstitute.org/) and Ensembl v.104 (http://may2021.archive.ensembl.org/index.html) were used for variant annotation.

## Human research participants

Policy information about studies involving human research participants and Sex and Gender in Research.

**Reporting on sex and gender**

Breakdown by genetically determined sex for the 100KGP discovery collection as provided in the Genomics England Research Environment: 40,332 female; 35,511 male; 1,696 not available.

**Population characteristics**

Collection of rare disease participants and relatives covering a wide range of pathologies. Breakdown by genetically determined most probable ancestry for the 100KGP discovery collection as provided in the Genomics England Research Environment: African: 2,762, Admixed American: 3,006; East Asian: 573; European: 63,493; South Asian: 7,705. Ages of 100KGP participants ranged between 0 and 110, with a lower quartile of 27, a median of 42 and an upper quartile of 58, with 18.4% under 18 overall.

**Recruitment**

Participants were identified by clinicians as eligible for recruitment to the 100,000 Genomes Project or for clinical testing through the United Kingdom's National Health Service Genomic Medicine Centres. The eligibility criteria are available from the Genomics England web site (https://www.genomicsengland.co.uk). Two cases were enrolled to the NIHR BioResource Rare Diseases (see https://doi.org/10.1038/s41586-020-2434-2 for more information on recruitment to that study). The studies were presented to eligible patients or their guardians by their clinicians widely across the health system, minimising selection bias subject to the enrolment criteria.

**Ethics oversight**

Participants of the 100KGP, the 100KGP Pilot Project and the GMS were enrolled to the National Genomic Research Library under a protocol approved by the East of England–Cambridge Central Research Ethics Committee (ref: 20/EE/0035). NBR participants were enrolled under a protocol approved by the East of England Cambridge South Research Ethics Committee (ref. 13/EE/0325). The Ethics Committee of University Hospitals Leuven approved genetic and experimental studies of a pedigree enrolled to the NBR in Belgium (ML3580/S50025 and S63666). Only participants who provided written informed consent for their data to be used for research were included in the analyses.

Note that full information on the approval of the study protocol must also be provided in the manuscript.

# Field-specific reporting

Please select the one below that is the best fit for your research. If you are not sure, read the appropriate sections before making your selection.

☒ Life sciences ☐ Behavioural & social sciences ☐ Ecological, evolutionary & environmental sciences

For a reference copy of the document with all sections, see nature.com/documents/nr-reporting-summary-flat.pdf

# Life sciences study design

All studies must disclose on these points even when the disclosure is negative.

**Sample size**

Statistical power to identify genetic associations with rare diseases depends on various factors including the sample sizes and genetic homogeneities of case groups. To our knowledge, a formal sample size calculation was not performed for the 100,000 Genomes Project. However, the study was informed by previous smaller studies showing sufficient power (see references in Turro et al. (2020), Nature).

**Data exclusions**

None.

**Replication**

Replication was achieved by analyzing genomes in the three collections other than the 100KGP discovery collection: the 100KGP Pilot project, the NIHR BioResource–Rare Diseases and the Genomic Medicine Service.

**Randomization**

Recruitment and genome sequencing were performed concurrently across rare disease categories, thus randomizing the order in which individuals were sequenced with respect to phenotype.

**Blinding**

This is an observational genetic study, not a clinical trial. As genome sequencing followed enrolment, participants and investigators were unaware of the participant genotypes generated by the 100KGP at enrolment.

# Reporting for specific materials, systems and methods

We require information from authors about some types of materials, experimental systems and methods used in many studies. Here, indicate whether each material, system or method listed is relevant to your study. If you are not sure if a list item applies to your research, read the appropriate section before selecting a response.

## Materials & experimental systems

| n/a | Involved in the study |
|---|---|
| ☒ | ☐ Antibodies |
| ☒ | ☐ Eukaryotic cell lines |
| ☒ | ☐ Palaeontology and archaeology |
| ☒ | ☐ Animals and other organisms |
| ☒ | ☐ Clinical data |
| ☒ | ☐ Dual use research of concern |

## Methods

| n/a | Involved in the study |
|---|---|
| ☒ | ☐ ChIP-seq |
| ☒ | ☐ Flow cytometry |
| ☒ | ☐ MRI-based neuroimaging |
